# Case Report: Keratoacanthoma and type I diabetes secondary to treatment with PM8001, a bifunctional fusion protein targeting TGF-β and PD-L1

**DOI:** 10.3389/fonc.2023.1046266

**Published:** 2023-08-01

**Authors:** Rongbin Qi, Hailing Xu, Xinyu Fu, Yingying Yu, Dongqing Lv, Yujing Li, Susu He

**Affiliations:** ^1^ Department of Respiratory Medicine, TaiZhou Hospital of Zhejiang Province Affiliated to Wenzhou Medical University, Linhai, Zhejiang, China; ^2^ Department of Gastroenterology, Taizhou Hospital, Wenzhou Medical University, Linhai, Zhejiang, China

**Keywords:** immune checkpoint inhibitors, immune-related adverse reactions, type I diabetes, keratoacanthoma, PM8001

## Abstract

Immune-related adverse reactions primarily involve the skin and the endocrine, digestive, and respiratory systems. In the endocrine system, these adverse effects mainly include hypophysitis, thyroiditis, hypoadrenalism, and rarely, diabetes mellitus. The most common symptoms in the skin are pruritus, rash, and infrequently, eruptive keratoacanthoma. Here, we report a case of a 67-year-old woman who developed eruptive keratoacanthoma of the skin 6 weeks after beginning treatment with a bispecific antibody (PM8001), targeting both programmed cell death receptor 1 and transforming growth factor β, as well as type I diabetes mellitus–induced ketoacidosis after 13 weeks. The type I diabetes appeared to stabilize after insulin treatment, and the keratoacanthoma gradually resolved after drug discontinuation. This case report describes a case of the effects of PM8001 immunotherapy on the endocrine glands and skin, together with a review of the relevant literature, and summarizes the different clinical characteristics of rare immune-related adverse events resulting from PM8001 immunotherapy to provide a reference for their early detection, diagnosis, and treatment.

## Introduction

Immunotherapy for treating malignant tumors has progressed significantly in recent years and has been found to be effective for the treatment of refractory or recurrent tumors. Immune checkpoint inhibitors (ICIs), including anti-programmed cell death receptor 1 (PD-1), ligand programmed cell death ligand 1 (PD-L1), and T lymphocyte–associated antigen 4 (CTLA4), can primarily exert their antitumor effects by reactivation of the killing function of effector T cells (1). Due to the unique antitumor mechanism of ICIs, their adverse effects differ from those of traditional radiotherapy, chemotherapy, and targeted therapy, all of which mainly cause dysfunction/damage throughout the body. Therefore, the adverse effects of ICIs are often referred to as immune-related adverse events (irAEs) (2).

Despite the significant success of ICIs in the treatment of cancer, many exhibit limited therapeutic responses. This has spurred the development of novel combination therapies, such as the use of ICIs combined with the targeting of transforming growth factor β (TGF-β). TGF-β signaling is one of the important mechanisms responsible for immunosuppression in the tumor microenvironment, and thus, TGF-β may enhance therapeutic resistance to ICIs in cancer patients (3). However, although this dual immune-targeting formulation enhances the body’s antitumor response, it can also induce abnormal immune-associated responses, resulting in serious adverse effects (4). In this study, we present a case of a patient with advanced non-small cell lung cancer (NSCLC) in which the cancer progressed after multiple lines of treatment with PM8001 (targeting PD-L1 and TGF-β) during the development of eruptive keratoacanthoma (KA) and type I diabetes mellitus (T1DM).

## Case report

**Figure 1:**
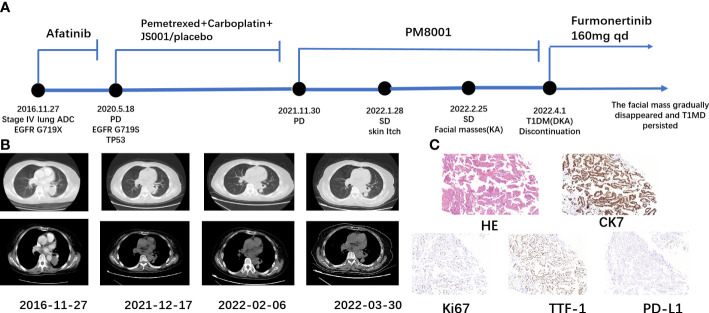
Schematic representation of the patient’s treatment history. **(A)** Treatment timeline. **(B)** Chest CT images throughout the disease course. **(C)** Pathological examination of the first lung tissue biopsy. Immunohistochemical testing (100×) results showed positive expression of CK7, Ki67, TTF-1, and PD-L1.

Here, we report a case of a 67-year-old woman who developed eruptive keratoacanthoma of the skin and T1DM-induced ketoacidosis after treatment with PM8001, a bispecific antibody targeting both PD-L1 and TGF-β (**Figure 1A**). Computed tomography (CT) on 27 November 2016 showed a left lower lung occupancy (**Figure 1B**). Bronchoscopic biopsy and immunohistochemical (IHC) analysis revealed adenocarcinoma with positive expression of TTF-1, CK7, PD-L1, and Ki67 (**Figure 1C**). Due to the rapid growth of the lesions, surgical treatment was not appropriate, and epidermal growth factor receptor (EGFR) genetic testing revealed EGFR (G719X). The patient received afatinib-targeted therapy for 3 years with regular routine blood and chest CT examinations. On 18 May 2020, chest CT re-examination indicated considerable tumor progression, and tissue needle biopsy suggested EGFR (G719S) and TP53-positive, T790M-negative. After further evaluation, the patient received a continuous regimen of “pemetrexed 762 mg + carboplatin 435 mg + JS001 (toripalimab)/placebo 240 mg D1” from 23 June 2020 to 19 November 2020. Tumor progression was detected on review (30 November 2021), but no obvious JS001/placebo-related adverse events occurred during the treatment. She was then enrolled in a phase I clinical trial to evaluate the tolerability, safety, and pharmacokinetic profile, as well as the preliminary efficacy, of PM8001 injection for patients with advanced tumors, and a phase IIa clinical trial to examine the preliminary efficacy in patients with advanced tumors (protocol number CPM8001-A001), which was conducted by Pumis Biotechnology (Zhuhai) Co., Ltd. The patient received continuous treatment with PM8001, administered intravenously at 1,066 mg (20 mg/kg) every 14 days per course, from 1 January 2022 to 25 March 2022.

**Figure 2:**
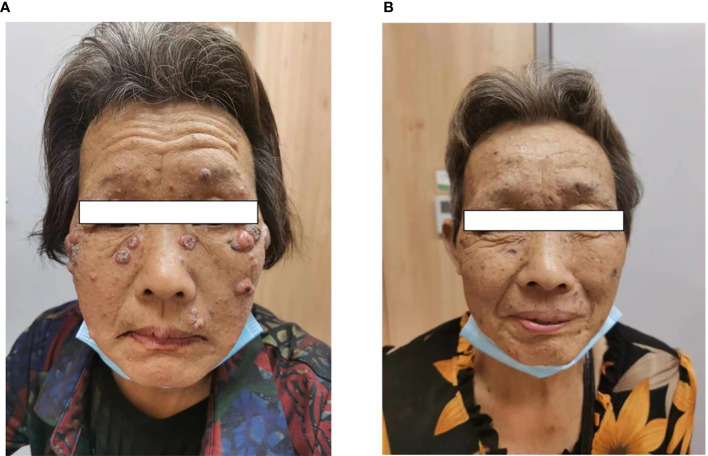
**(A)** Characterization of facial masses after immunotherapy in patients. **(B)** The patient experienced regression of the facial mass after discontinuation of the drug.

**Figure 3:**
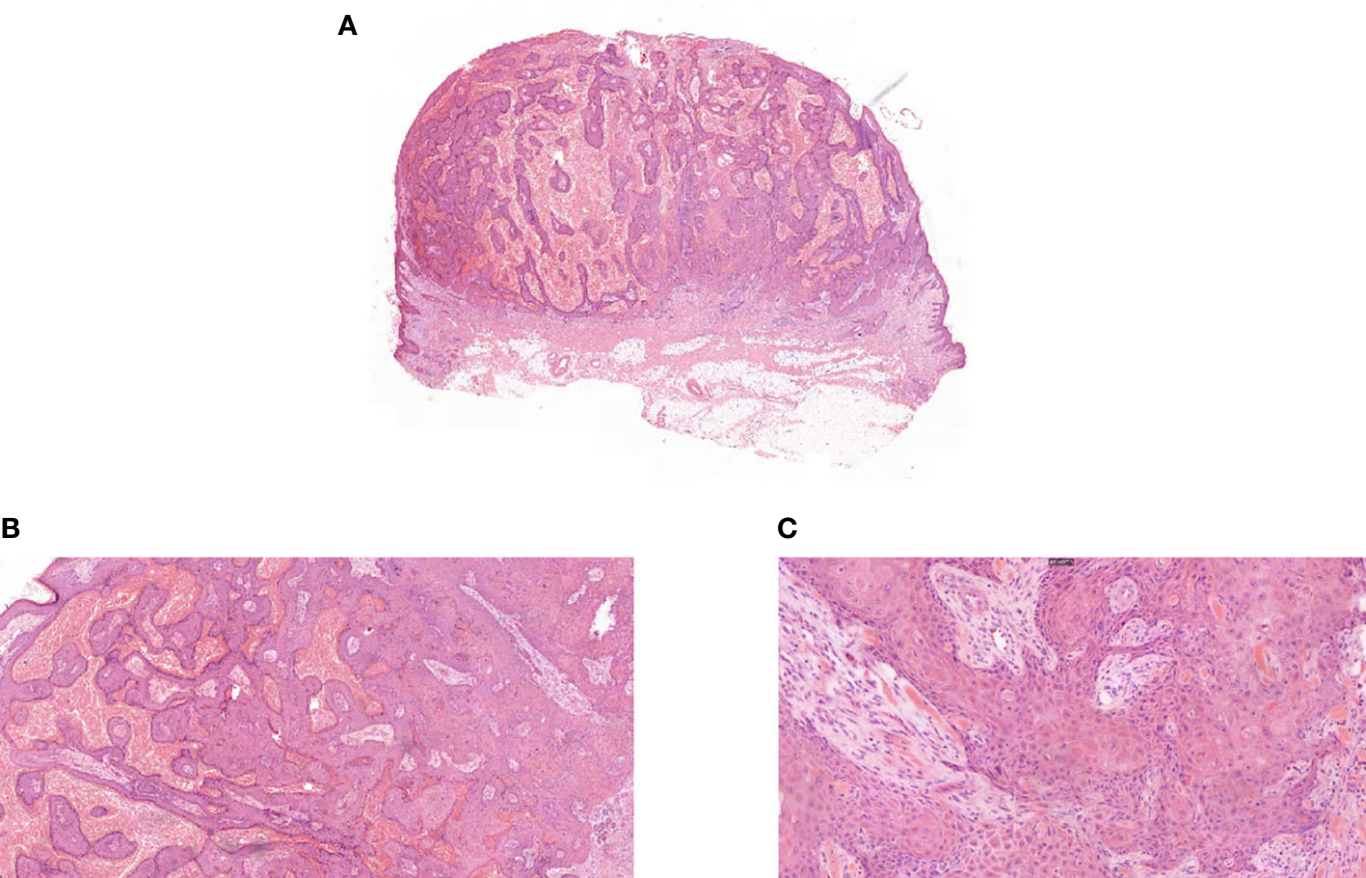
**(A)** Pathologic biopsy of facial mass ultralow power view. **(B)** Pathologic biopsy of facial mass magnification 50×. **(C)** Pathologic biopsy of facial mass magnification 200×.

During the fourth week of treatment (end of January 2022), the patient experienced generalized pruritus, which was initially treated with a series of medications such as Compound Camphor menthol liniment, loratadine, cetirizine hydrochloride, calamine zinc oxide liniment, Dened cream, and chlorpheniramine maleate. The symptoms initially stabilized. However, approximately 6 weeks after the start of treatment (February 2022), multiple facial masses appeared, with the largest one at the right eye angle measuring approximately 2 cm in diameter and protruding from the skin surface (**Figure 2A**). The skin texture was slightly hard, mobile, and had clear borders with a regular morphology but no tenderness, wave sense, obvious redness, or ulceration of the ruptured surface skin was observed, and no specific treatment was applied. The facial mass gradually increased in size over the next 2 weeks, and on 28 February 2022, a definite diagnosis of keratoacanthoma (KA) was made after local excision of the mass (**Figure 3**). The PM8001 was discontinued because of the severe immune adverse effects, and the facial KA eruptions gradually resolved (**Figure 2B**).

Thirteen weeks after the start of the PM8001 treatment, the patient presented with dry mouth, polydipsia, and polyuria without obvious triggers and that gradually worsened over time. On 1 April 2022, the patient was hospitalized due to a fasting glucose level of 33.38 mmol/l and suspected diabetic ketoacidosis (DKA). On admission, the patients showed fingertip blood ketone bodies of 4.8 mmol/L, fingertip blood glucose of 41.6 mmol/L, and markedly subnormal levels of C-peptide, so type 1 diabetes mellitus (T1DM) was initially considered. Additional examination findings are presented in **Table 1**.

**Table 1:** Laboratory results for the patient.

Variable	Value	Reference range
Biochemistry		
ALT (U/L)	20	13-69
AST (U/L)	19	13-35
K (mmol/L)	5.05	3.50-5.10
Na (mmol/L)	130	137-145
Cl (mmol/L)	94.4	98-107
urea (umol/L)	11.73	2.50-6.10
Creatinine (umol/L)	154	46-92
Diabetes-related examination		
Fingertip blood glucose (mmol/L)	41.6	4.1-5.9
Fingertip ketone bodies	4.8	<0.3mmol/L
HbA1c (%)	7.3	4.5-6.3
ICA	0.06	<1.00
GAD-Ab (IU/mL)	0.96	<10.00
IA	0.03	<1.00
TP-Ab (IU/mL)	<0.70	<10.00
Steamed bread meal experiment		
FBG (mmol/L)	6.36	3.89-6.11
GLU 2h (mmol/L)	8.09	4.40-7.80
C-peptide (ng/mL)	<0.03	0.78-5.19
C-peptide 2h (ng/mL)	<0.01	/
Urinalysis		
Urine glucose	2+	/
Urine ketone	negative	/
Urine protein	negative	/
Urine Occult Blood	+	/

ALT, alanine transaminase; AST, aspartate aminotransferase; HbA1c, hemoglobin A1c; ICA, islet cell antibody; GAD-Ab, glutamic acid decarboxylase antibody; IA, insulin antibody; TP-Ab, tyrosine phosphatase antibodies; FBG, fasting blood glucose; GLU, glucose.

## Discussion

The primary cause of irAEs induced by ICIs is the overstimulation of T lymphocytes leading to aberrant immune responses. These irAEs are commonly observed in the skin, endocrine system, digestive system, lungs, and other organs. The response rates for irAEs of any grade range from 30% to 5%–10% for grade ≥ 3, with mortality rates of up to 1% in some cases (5, 6). Endocrine-related irAEs often manifest as hypophysitis, hypoadrenalism, thyroiditis, and rarely, diabetes mellitus, particularly T1DM (7, 8).

A meta-analysis of serious adverse events in advanced NSCLC treated with ICIs showed that severe endocrine rAEs and dermatological irAEs were not entirely consistent across different immunotherapy regimens (9). It was found that various endocrine-related irAEs occurred mainly during treatment with PD-L1/PD-1 inhibitors but rarely during treatment with CTLA-4 inhibitors. However, dermatological irAEs were found mainly during treatment with PD-L1/PD-1 combined with CTLA-4 inhibitors. In the present case, the patient was treated with bispecific antibodies, targeting both PD-L1 and TGF-β, and an outbreak of severe cutaneous and endocrine irAEs was observed after the treatment.

Fulminant KAs have been defined as multiple KAs occurring within a short period. In the present case, we describe the case of a patient treated with PM8001 who developed multiple hyperkeratotic papules on her face after 8 weeks of treatment. These were initially suspected to be high-grade squamous cell carcinomas (SCCs), while the pathological biopsy indicated KA. In theory, SCC is not considered a side effect of PD-1 inhibitors, which promote immune surveillance rather than blocking or shunting proliferation-associated pathways (10, 11). KA is a subtype of SCC typically considered to have a more benign course, with spontaneous regression over several months (12, 13). However, KA has been reported in patients treated with ICIs, including PD-1 inhibitors such as nivolumab and pembrolizumab (14, 15). In a recent case, a patient treated with pembrolizumab developed multiple skin lesions with pseudo-epithelioma-like hyperplasia during treatment and for approximately 2 months after treatment discontinuation which clinically and pathologically resembled early invasive SCC or KA. Distinguishing between the two can be challenging, but histopathological evaluation and close clinical follow-up can help differentiate them (15). Histopathological features suggestive of KA include the presence of crater-like structures, symmetric overall pushing growth patterns, distinct demarcation from the surrounding stroma, abundant glassy cytoplasm, and intraepithelial elastin fibers. SCC, on the other hand, may exhibit a rudimentary growth pattern with abnormal forms of mitotic activity, marked cytologic atypia, acantholysis, and paradoxical maturation (16). Furthermore, relying solely on histopathological findings to predict the biological behavior of a lesion may not be sufficient, particularly if only a small portion of the biopsy is obtained. Therefore, it is crucial to conduct close clinical monitoring. Based on pathological examination, the patient described in this study was diagnosed with KA but the presence of higher-than-normal levels of carcinoembryonic antigen indicates that the possibility of malignant transformation cannot be entirely ruled out.

T1DM is a relatively rare irAE that occurs more commonly in patients treated with PD-1 inhibitors, occasionally in patients treated with PD-L1 inhibitors, and essentially not at all in patients treated with CTLA-4 inhibitors (17). PD-1 is widely expressed in immune tissues and various target organs (such as pancreatic B cells). Targeted blockade of PD-1 and PD-L1 interactions using pharmacological agents could effectively stimulate T lymphocyte activation with vas, thus leading to the destruction of pancreatic B cells, which provides a rationale for anti-PD-L1-induced T1DM (18). A recent study showed that the median onset of T1DM induced by ICIs was 20 weeks, and most patients treated with PD-1/PD-L1 monotherapy usually developed the disease after 10 weeks (17, 19). Several preclinical studies have found that the combination of anti-PD-L1 and TGF-β antibodies can significantly reduce TGF-β signaling in the stromal cells and stimulate T-cell entry into the tumor, thus leading to tumor suppression (20). TGF-β has multiple actions, including inducing the generation of regulatory T cells (21). Regulatory T cells modulate tolerance in the organism and have inhibitory effects on B-cell activity. Thus, the combined administration of anti-PD-L1 and anti-TGF-β antibodies can markedly increase the likelihood of developing T1DM. In addition, combining PD-L1 blockade and TGF-β sequestration can adversely affect the pancreas and thus might promote the development of T1DM.

ICI-associated diabetes has an acute onset and rapid progression, and the symptoms can appear quickly with hyperglycemia or symptoms of DKA. According to the latest European-American guidelines, it is recommended that the patients receive regular monitoring of their blood glucose levels during immunotherapy to detect the presence or absence of type 1 or type 2 diabetes (2, 22–25). However, it is difficult to predict the occurrence of diabetes mellitus even with regular monitoring of the blood glucose changes. In general, this is indicated when insulin and C-peptide levels fall below one-third of normal within a short period after the onset of immune-related diabetes, and neither of the measures returns to normal (26). The C-peptide levels in the patient in this case were also significantly decreased to less than one-third of normal and did not recover in the long term; thus, we considered that dynamic monitoring of changes in the C-peptide levels during PM8001 treatment might be an important strategy for predicting immune-related T1DM. In addition, recent studies have also found that while diabetes-associated antibodies are not associated with the onset of immune-related diabetes, they might influence its duration (17). It was found that patients with antibody-positive ICI-related T1DM show rapid onset of diabetes and a high incidence of DKA. However, there were no obvious abnormalities in the patient’s diabetes-related autoantibody levels; the onset was rapid and included DKA, which was somewhat different from other cases. This could potentially be related to the dual immune attack of anti-PD-L1 combined with anti-TGF-β on the pancreas. Overall, these observations suggest that clinicians need to regularly monitor the various indices related to islet function during the application of combination therapy with ICIs, with especially close monitoring of blood glucose and islet function for 10 weeks during the application of immunotherapy, as well as informing patients of the risk of developing complications such as diabetes to avoid the occurrence of high-risk irAEs.

## Conclusion

ICIS are widely used in malignancies and have significantly prolonged the survival of cancer patients. However, we still need to pay attention to the occurrence of immune-related adverse reactions while focusing on their efficacy, in which the pathogenesis of endocrine- and skin-associated irAEs is not well defined and the clinical presentation is variable and may be life-threatening for severe cases. In particular, the incidence of irAEs may be increased during the combination of immunotherapies, particularly for blockade of PD‐L1 and TGFβ targeting immunosuppressive drugs such as pm8001, and patients can experience multisystem irAEs, such as endocrine and cutaneous. Therefore, close attention should be paid to the endocrine system–related indicators during the medication process. Regular observation of changes of skin malignancy and other life-threatening serious irAEs needs to be considered months after the end of the medication. Overall, prevention of the onset of life-threatening serious irAEs during and even after completion of the treatment should be carefully monitored for an optimal therapeutic effect.

## Data availability statement

The original contributions presented in the study are included in the article/supplementary material. Further inquiries can be directed to the corresponding author.

## Ethics statement

Written informed consent was obtained from the individual(s) for the publication of any potentially identifiable images or data included in this article.

## Author contributions

RQ and HX: data extraction and article writing. XF and YY: data collection and article writing. SH and DL: expert opinion. All authors contributed to the article and approved the submitted version.
